# Association of Genetic Variants in *PNPLA3*, *MBOAT7*, and *MARC1* with Metabolic and Hematological Immune-Inflammation Indices in Adolescent Females with and Without MASLD: A Multilevel Risk-Allele Burden Analysis

**DOI:** 10.3390/ijms27114837

**Published:** 2026-05-27

**Authors:** Simona Jurkovic Mlakar, Aleksandra Klisic, Janja Marc, Barbara Ostanek

**Affiliations:** 1 Department of Clinical Biochemistry, Faculty of Pharmacy, University of Ljubljana, 1000 Ljubljana, Slovenia; simona.jurkovicmlakar@ffa.uni-lj.si (S.J.M.); janja.marc@ffa.uni-lj.si (J.M.); 2 Faculty of Medicine, University of Montenegro, 81000 Podgorica, Montenegro; 3 Center for Laboratory Diagnostics, Primary Health Care Centre, 81000 Podgorica, Montenegro

**Keywords:** adolescence, MASLD, metabolic inflammation, *MARC1*, *MBOAT7*, *PNPLA3*, SII, TyG

## Abstract

Metabolic dysfunction-associated steatotic liver disease (MASLD) is increasingly recognized during adolescence and exhibits sex-specific characteristics. Its prevalence in late adolescent females is around 10% in the general population and exceeds 30% in obesity. Genetic variants in *PNPLA3*, *MBOAT7*, and *MARC1* modulate hepatic fat accumulation and liver injury in adults, but evidence in adolescent females remains limited. This study examined MASLD-related variants (*PNPLA3* rs738409, *MBOAT7* rs641738, *MARC1* rs2642438) in relation to metabolic and immune-inflammation indices in a late-adolescent female cohort. A cross-sectional analysis was performed in a prospectively assembled female cohort (*n* = 150; age 16–19 years). Ultrasound-defined MASLD prevalence was 16.7%. Although genotype-wise differences did not reach statistical significance, MASLD prevalence was directionally higher among *PNPLA3* G- and *MBOAT7* T-allele carriers, while a non-uniform, directionally favorable pattern was observed for the *MARC1* A allele. Nominal, unadjusted differences were observed for low-density lipoprotein cholesterol (LDL-c) and systemic immune-inflammation index (SII) across *MBOAT7 genotypes*. Cumulative risk-allele burden analyses identified nominal trends for triglycerides (K-W *p* = 0.05; J-T *p* = 0.014) and triglyceride-glucose (TyG) index (K-W *p* = 0.034; J-T *p* = 0.008), which were not retained after adjustment for age and body mass index. Overall, these findings indicate modest, exploratory genotype-related patterns in metabolic and hematological immune-inflammation indices within a relatively healthy, late-adolescent female population. TyG and SII exhibited substantial inter-individual variability but did not demonstrate independent predictive value. Larger, longitudinal studies with advanced imaging are required to clarify the role of genetic variation and simple hematological metabolic-inflammation indices in early MASLD risk assessment in adolescent females.

## 1. Introduction

Metabolic dysfunction-associated steatotic liver disease (MASLD), previously termed non-alcoholic fatty liver disease (NAFLD), is increasingly recognized during childhood and adolescence and exhibits several sex-specific features that are particularly relevant for girls and young women. Although MASLD affects both sexes, female-specific physiological determinants, including pubertal hormonal transitions, patterns of insulin-resistance, adipose-tissue distribution, and inflammatory responses, shape susceptibility and may influence the expression of genetics during adolescence. These interrelated axes influence hepatic lipid handling and may modify the penetrance of common MASLD-associated variants in late-adolescent females [1,2,3,4].

In pediatric populations, MASLD prevalence among late-adolescent females is estimated at approximately 10% in general cohorts and rises substantially (up to 39%) in those with overweight or obesity, paralleling global trends in adolescent adiposity and metabolic dysfunction [5]. Prevalence is even higher in young females with early-onset type 2 diabetes, where approximately one-third are affected. Given projected increases in adolescent overweight and obesity through 2030 and beyond, early identification of at-risk adolescent females is a public-health priority, underscoring the need for reliable, non-invasive metabolic markers. Several sex-linked physiological mechanisms likely contribute to MASLD pathogenesis in females. Pubertal insulin resistance tends to be more pronounced in late-adolescent females, predisposing them to earlier impairments in glucose and lipid metabolism. Fluctuations in sex hormones, particularly relative reductions in estradiol, can diminish hepatic lipid oxidation and facilitate steatosis. In addition, obesity in females is frequently accompanied by dyslipidemia and increased systemic inflammation, both of which may accelerate MASLD progression. Moreover, female-predominant adipose-tissue patterns, characterized by substantial subcutaneous yet metabolically active adipose tissue depots, may further modulate lipid flux and inflammatory signaling relevant to hepatic health [6,7,8,9,10].

Genetic susceptibility also contributes to MASLD risk in youth. Among pediatric cohorts, the *PNPLA3* I148M (rs738409) variant consistently represents the strongest determinant of hepatic steatosis. Studies in children and adolescents, including those by Nobili et al. [11], Stasinou et al. [12], and Mansoor et al. [13] reported markedly higher frequencies of *PNPLA3* risk genotypes in MASLD compared with controls. Protective effects of *HSD17B13* and *MARC1* and fibrosis-related associations with *SOD2* rs4880 have also been described [14], whereas evidence for *TM6SF2* in pediatric populations remains inconsistent. Collectively, pediatric data converge on *PNPLA3* as the dominant genetic risk locus, with *MARC1* A-allele commonly protective and *SOD2* more closely associated with fibrosis-related oxidative pathways.

However, many pediatric studies enrolled broad age ranges (typically 5–18 years), included both sexes and predominantly patients with advanced MASLD (especially fibrosis). Consequently, the genetic architecture of early, subclinical susceptibility in biologically homogeneous, female late-adolescent populations remains poorly defined (Table S1).

When studies are not sex-stratified, mechanistic and epidemiologic evidence must be interpreted in the context of female physiology. *PNPLA3* is the most powerful genetic determinant of MASLD in women: the I148M variant promotes hepatic triglyceride accumulation, impairs lipid-droplet remodeling, and increases the risk of progression to NASH and fibrosis [15].

Mechanistic further indicates that *MBOAT7* deficiency alters phosphatidylinositol remodeling, thereby promoting hepatic lipid accumulation and increasing fibrosis risk. These mechanisms are applicable to late-adolescent females, particularly under conditions of insulin resistance or obesity [16]. In contrast, the *MARC1* A allele tends to be protective, with carriers exhibiting lower aminotransferases and reduced hepatic steatosis severity across human cohorts [17]. These findings are consistent across human cohorts and support *MARC1*’s favorable impact on female metabolic liver health.

From a biomarker perspective, two readily accessible indices map closely onto biological pathways relevant for female MASLD. The triglyceride–glucose (TyG) index serves as a widely validated surrogate for insulin resistance, a key driver of adolescent MASLD risk [18], whereas the systemic immune-inflammation index (SII), integrating neutrophil, platelet, and lymphocyte counts, reflects inflammation-driven metabolic stress and may interact with genetic predisposition in late-adolescent females [19]. Both indices are inexpensive, reproducible, and clinically practical tools for early metabolic risk stratification.

Prior pediatric MASLD genetic studies [11,12,13,14] typically enrolled both sexes, spanned broad age ranges (often 5–18 years), had substantially higher proportions of obesity and MASLD, and frequently emphasized advanced disease (especially fibrosis). To our knowledge, no study has specifically investigated a female-only, late-adolescent (16–19 years), biologically homogeneous cohort with relatively lower obesity burden to interrogate early (subclinical) susceptibility. Addressing this gap may facilitate early identification of adolescent girls at increased cardiometabolic risk.

We therefore hypothesized that: (a) risk-alleles in *PNPLA3* and *MBOAT7* would be associated with adverse metabolic (e.g., higher triglycerides, elevated TyG) and hematological immune-inflammation (e.g., higher SII, neutrophilia) profiles in adolescent females, whereas the *MARC1* A-allele confers protective effects, with the G-allele representing the non-protective state; (b) a higher cumulative three-locus genetic risk-allele burden (*PNPLA3* G + *MBOAT7* T + *MARC1* G) would be associated with monotonic worsening of metabolic and inflammatory markers; and (c) TyG and SII would vary across genetic strata and MASLD status, reflecting metabolic and hematological immune-inflammatory heterogeneity during late adolescence.

## 2. Results

### 2.1. Cohort Overview

A total of 150 adolescent females aged 16–19 years (mean ± SD: 16.67 ± 0.86) were included. MASLD was present in 16.7% (25/150). Baseline anthropometric, biochemical, and hematologic characteristics are presented in Table 1. Definitions of metabolic and inflammatory indices assessed in this study are presented in Table S2.

Overall, participants represented a relatively healthy, lower-obesity cohort, with liver-enzyme and lipid values largely within expected ranges for this age group. Mean liver-enzyme values were within low-to-moderate ranges (ALT 17.75 ± 7.70 U/L; AST 20.29 ± 5.57 U/L). The TyG index averaged 10.66 ± 0.69 (*n* = 144), while the SII demonstrated substantial inter-individual variability (mean 631.74 ± 314.72), reflecting heterogeneous metabolic and inflammatory states typical of adolescence.

### 2.2. Genotype Distribution

**Figure 1:**
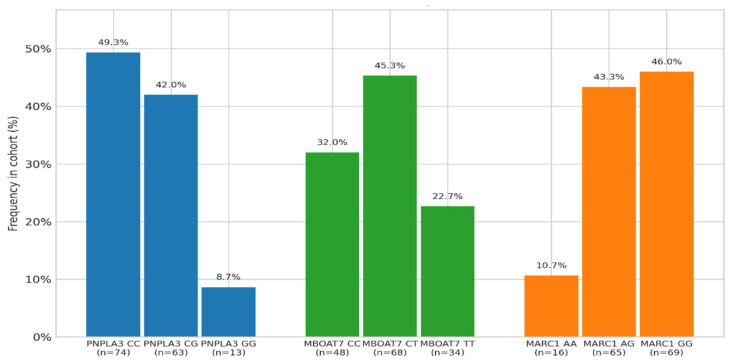
Genotype frequencies in late-adolescent females (*n* = 150): *PNPLA3*, *MBOAT7*, *MARC1*.

Genotype frequencies for the three variants of interest showed expected distributions in late-adolescent females (Figure 1). Genotype distributions conformed to Hardy–Weinberg equilibrium (*PNPLA3*, *p* = 0.94; *MBOAT7*, *p* = 0.30; *MARC1*, *p* = 0.91). *PNPLA3* rs738409 risk-allele carriage was common (CG 42.0%, GG 8.7%). For *MBOAT7* rs641738, the CT genotype was most frequent (45.3%). For *MARC1* rs2642438, AG and GG genotypes together accounted for nearly 90% of participants.

In unadjusted exploratory analyses using Kruskal–Wallis and Jonckheere–Terpstra tests, no genotype-wise differences reached statistical significance for *PNPLA3* across ALT, AST, GGT, TG, HDL-c, LDL-c, TyG, or SII (all *p* ≥ 0.31), and none for *MARC1* (*p* ≥ 0.28). In contrast, *MBOAT7* showed nominal group differences for LDL-c and SII (*p* = 0.05), driven by relatively higher values in TT versus CC/CT genotypes.

### 2.3. Genotype–Phenotype Comparisons (Biochemical and Index Traits)

For each of the three variants (*PNPLA3*, *MBOAT7*, *MARC1*), we compared biochemical markers (ALT, AST, GGT, TG, HDL-c, LDL-c) and derived indices (TyG and SII) across genotype groups (Table 2; Table S3).

In unadjusted exploratory analyses (Kruskal–Wallis), no significant differences were observed across *PNPLA3* genotypes for any trait (all *p* values of ≥ 0.31). For the *MARC1* variant, no significant differences were detected (*p* ≥ 0.28). For the *MBOAT7* variant, TyG, TG, LDL-c and SII showed nominal group differences (*p* values of 0.08, 0.12, 0.05 and 0.14, respectively), driven by relatively higher values in TT versus CC/CT genotypes (Table 2), showing trend-level in JT tests (respective *p*-values of 0.01, 0.02, 0.10 and 0.10). After adjustment for age and body mass index (BMI) in linear regression models for continuous metabolic and inflammatory traits, no statistically significant associations were observed (Table S3).

### 2.4. MASLD Prevalence Across Genotype Groups

**Figure 2:**
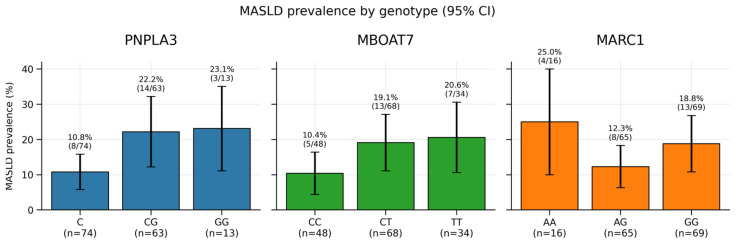
Prevalence of MASLD (%) by genotype with 95% Wilson confidence intervals. For each gene, bars show MASLD prevalence within genotype groups; numbers above the bars indicate the exact proportion (*x/n*) and percent. Wilson 95% CIs are used to provide robust coverage with modest stratum sizes. Patterns are directionally consistent with prior pediatric/adolescent literature for *PNPLA3* and *MBOAT7* risk-alleles and with the protective *MARC1* A165T signal.

In line with prior evidence, MASLD prevalence was higher among *PNPLA3* G- and *MBOAT7* T-allele carriers, whereas a directionally protective pattern was observed for MARC1 A165T (see Figure 2). For transparency, given modest genotype strata (e.g., *PNPLA3* GG; *MARC1* AA), we present 95% Wilson CIs. Data are presented in Figure 2.

Within *PNPLA3* (rs738409), MASLD prevalence was higher in G-allele carriers (CG 22.2%; GG 23.1%) than in CC (10.8%). *MBOAT7* groups showed a similar trend, with T-allele carriers showing higher prevalence (CT 19.1%; TT 20.6%) than CC (10.4%). For MARC1 rs2642438, MASLD prevalence followed a non-monotonic pattern, with the AG genotype showing the lowest prevalence (12.3%) and the AA genotype the highest (25.0%). This distribution does not support a simple additive ‘A-allele protective’ model. Given the small size of the AA subgroup (*n* = 16), these findings should be interpreted cautiously and suggest a more complex or context-dependent effect rather than uniform protection (Figure 2). No significant association with MASLD was observed for *MBOAT7* rs641738, *MARC1* rs2642438, or *PNPLA3* rs738409 in multivariable logistic regression models adjusted for age and BMI (Table 3).

### 2.5. Risk-Burden Analysis (Three-Locus Model)

**Figure 3:**
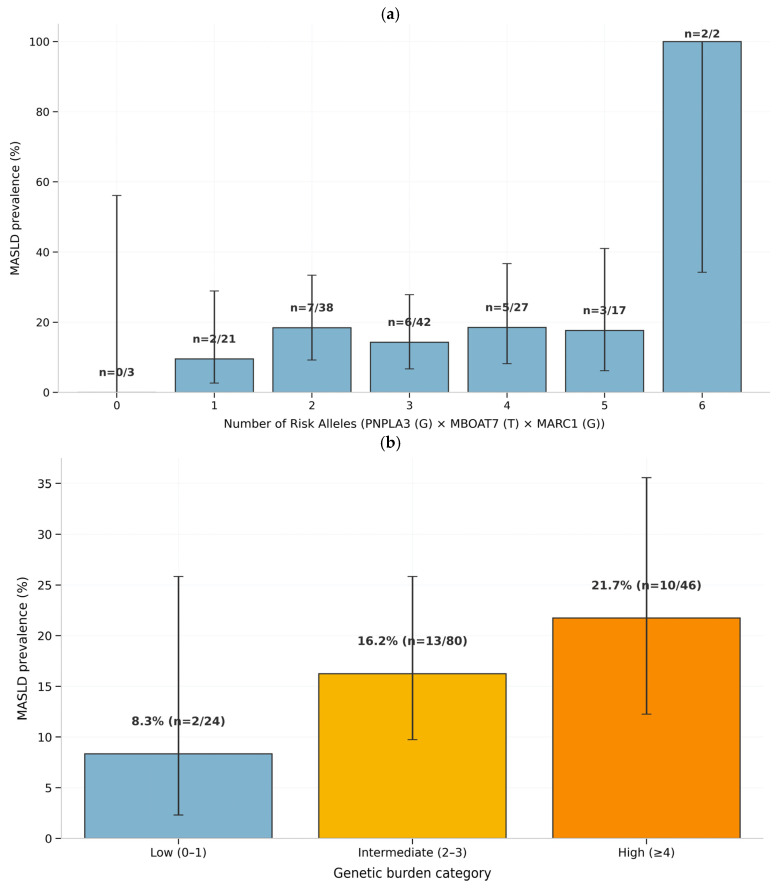
(**a**) Prevalence of MASLD shown across the full 0–6 range of the cumulative genetic risk-allele genetic burden calculated from *PNPLA3* (G), *MBOAT7* (T) and *MARC1* (G). Bars show prevalence (%), error bars represent Wilson 95% confidence intervals, and annotations display *n* = MASLD cases/total per burden level. Extreme burden categories contain very small numbers of participants and are shown for completeness only. (**b**) Prevalence of MASLD by cumulative genetic burden category. Genetic burden categories were defined as Low: 0–1 risk-alleles, Intermediate: 2–3 risk-alleles, and High: ≥4 risk-alleles based on the combined burden of *PNPLA3* (G), *MBOAT7* (T), and *MARC1* (G). Bars show MASLD prevalence (%), error bars represent 95% Wilson confidence intervals, and labels indicate the number of MASLD cases over the total number of participants in each category (*n* = cases/total). Categories are shown descriptively and should not be interpreted as evidence of monotonic genetic effects.

Our results showed that the cohort exhibited modest liver-enzyme levels and substantial inter-individual variability in TyG and SII. *PNPLA3* and *MBOAT7* risk-allele carriage was frequent. Using a three-locus cumulative genetic burden score (*PNPLA3* G + *MBOAT7* T + *MARC1* coding per specification), the nominal trends were observed for Triglycerides (Kruskal–Wallis *p* = 0.050; JT *p* = 0.014) and TyG (Kruskal–Wallis *p* = 0.034; JT *p* = 0.008) (Figures S1 and S2). These associations were not retained after adjustment for age and BMI. MASLD prevalence was higher among *PNPLA3* G-allele and *MBOAT7* T-allele carriers, while *MARC1* A165T showed a lower prevalence pattern. Cumulative burden analysis demonstrated increasing MASLD prevalence across higher allele-count categories (Figure 3). Extreme burden categories contain very small numbers of participants and are shown for completeness only.

Extreme burden categories included very small sample sizes (e.g., *n* = 2), resulting in unstable prevalence estimates. Accordingly, confidence intervals are wide, and these categories should not be interpreted as evidence of monotonic genetic effects. The burden analysis is therefore considered descriptive and hypothesis-generating.

## 3. Discussion

In this female-only cohort of 150 late-adolescent participants, the prevalence of MASLD was 16.7%, consistent with epidemiologic estimates of approximately 10–20% in unselected youth [20,21]. Metabolic parameters, including liver enzymes, lipids, TyG, and SII, were largely within expected ranges for healthy adolescents yet demonstrated notable inter-individual variability [22,23]. These findings support the concept that adolescent MASLD reflects early interactions between genetic susceptibility, insulin-resistance pathways, and inflammatory activity, often preceding overt biochemical abnormalities [24].

This study provides two key contributions to pediatric MASLD genetics. First, by focusing exclusively on late-adolescent females (16–19 years), it captures a biologically homogeneous group, reducing variability related to puberty, hormonal fluctuations, and sex-specific metabolic changes. Second, it integrates three biologically plausible MASLD loci (*PNPLA3*, *MBOAT7*, *MARC1*) with accessible indices (TyG, SII), offering a combined framework for assessing early metabolic and inflammatory susceptibility. Unlike previous pediatric studies with mixed-sex, broad age cohorts (5–18 years), higher obesity prevalence and a greater focus on advanced disease (e.g., fibrosis), this study emphasizes early susceptibility in a well-defined and healthier female population.

Consistent with extensive literature, the *PNPLA3* I148M (rs738409) risk-allele showed directional association with hepatic steatosis, with nearly two-fold MASLD prevalence among G-allele carriers compared with CC homozygotes (22–23% vs. 11%). However, no significant differences were observed in biochemical markers (ALT and lipids) and indices (TyG, SII) across *PNPLA3* genotypes, supporting prior pediatric imaging-based reports that the hepatic fat phenotype can emerge in carriers without detectable changes in circulating biomarkers, particularly at early disease stages [13,25].

The *MBOAT7* rs641738 T-allele demonstrated a similar directional pattern, with higher MASLD prevalence among T-allele carriers (19–21% vs. 10% in CC) and nominal group differences for LDL-c and SII (TT > CC/CT). These observations are consistent with proposed roles for *MBOAT7* in phosphatidylinositol remodeling, steatosis, and inflammation-linked progression, although population-specific variability is well recognized. Larger, female-focused cohorts will be needed to confirm these preliminary observations and to disentangle metabolic from inflammatory contributors [5].

The *MARC1* A165T variant, widely regarded as protective in adults, showed a non-monotonic pattern, with the AG genotype showing the lowest MASLD prevalence (12.3%), and the highest in the AA genotype (25.0%). Biochemical parameters were otherwise similar across genotypes, which is plausible in a metabolically healthy, early-stage adolescent setting where protection may be most visible in steatosis prevalence rather than routine blood tests [26,27,28], particularly when disease is mild and variance in biochemical markers is limited. The effect of MARC1 may therefore be context-dependent and potentially modified by the presence of stronger risk-alleles, particularly in PNPLA3 and MBOAT7.

Taken together, the directional gradients of *PNPLA3*, *MBOAT7* risk-allele carriers for the MASLD prevalence suggest modest, directionally consistent variability in late-adolescent females, while largely normal-range biochemistry suggests that hepatic genetic effects can precede changes in routine serum markers.

When we combined *PNPLA3* (G), *MBOAT7* (T) and *MARC1* (G) -alleles into an unweighted 0–6 -allele count, we observed monotonic, nominal trends toward higher triglycerides and TyG with increasing risk-allele burden (Triglycerides: K–W *p* = 0.050; JT one-sided *p* = 0.014; TyG: K–W *p* = 0.034; JT one-sided *p* = 0.008). These gradients are biologically plausible as *PNPLA3* I148M and *MBOAT7* rs641738 risk-alleles have additive effects on hepatic fat and histological severity in adults, while the *MARC1* p.A165T variant is directionally protective. However, our higher-allele strata were relatively small and the unweighted score may dilute locus-specific effect sizes. Future studies should test weighted genetic risk scores (GRS) and validate findings using quantitative hepatic fat modalities (MRI-PDFF or quantitative ultrasound) to better resolve aggregate genetic effects on early metabolic indices.

Regarding early-risk indices, the cohort mean TyG (10.66) was typical for adolescence, and genotype associations were minimal, suggesting that genetic risk and insulin resistance may contribute additively rather than interactively at this early stage. In our group, SII reflected a generally low-grade inflammatory burden overall (mean 632) yet displayed nominal elevation in *MBOAT7* TT carriers, supporting mechanistic links between *MBOAT7* function and inflammatory signaling [5,23,29,30]. Together, these patterns indicate that TyG and SII capture inter-individual metabolic and hematologic immune-inflammatory variability in late adolescence, without demonstrating independent predictive utility for MASLD in this cohort.

Synthesizing these findings, we propose a working model in which genetic susceptibility (*PNPLA3*, *MBOAT7*) interacts with insulin resistance (TyG) and inflammatory tone (SII) to shape early MASLD risk in adolescent females, whereas *MARC1* does not appear to follow a simple additive protective pattern in this cohort. The modest effect sizes and near normal-range biochemistry observed here are consistent with subclinical disease stages and the recognized phenomenon whereby hepatic genetic risk can precede or exceed changes in standard blood-based markers.

A key strength of this study is the purposefully female-only, late-adolescent cohort and the integrated analysis of three biologically plausible loci with low-cost indices. Contemporary clinical pathways for steatotic liver disease prioritize non-invasive assessment, including serologic scores (e.g., FIB-4), ultrasound for steatosis, and elastography/ELF for fibrosis, particularly in populations with cardiometabolic risk. Our inclusion of TyG (insulin-resistance proxy) and SII (systemic immune-inflammation index) aligns with the metabolic-inflammatory framework of MASLD and with emerging pediatric data on their diagnostic and prognostic relevance [31].

The main limitations of the study include the cross-sectional design, modest genotype-stratum sizes for some contrasts (e.g., *PNPLA3* GG; *MARC1* AA). MASLD was defined using abdominal ultrasound in combination with cardiometabolic risk factors. While ultrasound is widely used in population-based studies, it is less sensitive for detecting low hepatic fat fractions compared with MRI-PDFF. This limitation is particularly relevant in an early or subclinical adolescent population and may have led to under-ascertainment of mild steatosis. Consequently, genetic associations with early hepatic fat accumulation may be underestimated in this study.

## 4. Materials and Methods

### 4.1. The Study Design, Population and Ethics

We conducted a cross-sectional analysis of a prospectively collected single-center adolescent cohort. The analytic sample was restricted to female participants aged 16–19 years, deliberately focusing on a narrow, late-adolescent window to reduce pubertal/hormonal heterogeneity and to emphasize early (subclinical) susceptibility rather than advanced disease. Available variables included anthropometry, fasting biochemistry, complete blood counts, lipid and liver panels, ultrasound-based hepatic steatosis status, and genotypes for three MASLD-related polymorphisms: *PNPLA3* rs738409 (C>G; I148M), *MBOAT7* rs641738 (C>T), and *MARC1* rs2642438 (G>A; A165T). These variants were selected based on robust prior evidence implicating them in MASLD susceptibility (*PNPLA3*, *MBOAT7*) or protection (*MARC1*) in human studies. For *MARC1*, the A-allele is known to be protective; therefore, for consistency with the risk-allele framework, the G-allele (non-protective) was included in the cumulative risk-score.

### 4.2. Laboratory Measures and MASLD Ascertainment

All laboratory parameters were obtained from fasting morning venous blood samples using routine clinical chemistry methods. Serum glucose, lipid fractions (triglycerides, HDL-cholesterol, LDL-cholesterol), and liver enzymes (alanine aminotransferase [ALT], aspartate aminotransferase [AST], and gamma-glutamyl transferase [GGT]) were measured using enzymatic, enzyme-coupled kinetic spectrophotometric assays performed on an automated clinical chemistry analyzer, i.e., Roche Cobas c503 chemistry analyzer (Roche Diagnostics GmbH, Mannheim, Germany). ALT and AST activities were determined according to International Federation of Clinical Chemistry (IFCC) reference methods, using NADH-linked reactions with continuous monitoring of absorbance decrease at 340 nm. GGT activity was assessed using a kinetic colorimetric method based on substrate conversion and spectrophotometric detection. All enzyme measurements were quantified by rate-of-change (kinetic) analysis, ensuring high analytical sensitivity and reproducibility.

Complete blood counts, including platelets and leukocyte subpopulations (neutrophils, lymphocytes, monocytes, eosinophils, basophils), were analyzed using an automated hematology analyser, i.e., Sysmex XN-1000 analyser (Sysmex Corporation, Kobe, Japan), according to standard laboratory protocols.

Alkaline phosphatase (ALP) was not routinely measured in this adolescent cohort. ALP primarily reflects cholestatic liver disease and bone turnover and does not provide relevant diagnostic information for early MASLD, which is characterized predominantly by hepatocellular steatosis rather than cholestasis. Therefore, ALP was not included in the biochemical characterization.

An abdominal ultrasound was performed by trained personnel using standard clinical ultrasound equipment as part of routine evaluation. Ultrasound is widely recommended as a first-line imaging modality for the detection of hepatic steatosis in pediatric and adolescent populations, due to its non-invasive nature, absence of ionizing radiation, accessibility, and suitability for epidemiologic studies. Current pediatric guidelines recognize ultrasound as the preferred initial imaging tool for MASLD detection, while fibrosis risk assessment typically relies on sequential serologic and advanced imaging approaches when clinically indicated [32,33].

MASLD was diagnosed by the presence of ultrasound-detected hepatic steatosis combined with at least one cardiometabolic risk factor, consistent with contemporary non-invasive diagnostic criteria and pediatric epidemiologic standards [1]:

1. BMI ≥ 25 kg/m^2^ or WC ≥ 94/80 cm for adolescent boys and girls, respectively.
2. Fasting serum glucose ≥ 5.6 mmol/L, or type 2 diabetes mellitus, or treatment for type 2 diabetes mellitus.
3. Systolic blood pressure ≥ 130 mm Hg, or diastolic blood pressure ≥ 85 mmHg.
4. Serum HDL-C ≤ 1.0 mmol/L for adolescent boys and HDL-C ≤ 1.30 mmol/L) for adolescent girls, respectively.
5. Serum TG ≥ 1.70 mmol/L.

### 4.3. Genetic Polymorphisms and Quality Control

Genomic DNA was isolated from peripheral blood leukocytes by using a FlexiGene DNA kit (Qiagen, Hilden, Germany). Polymorphisms in *PNPLA3* rs738409, *MBOAT7* rs641738, and *MARC1* rs2642438 gene were genotyped using validated TaqMan Assays (C__7241_10, C__8716820_10 and C___12357721_10 respectively), Thermo Fischer Scientific, Waltham, MA, USA) in LightCycler^®^ 480 Instrument II (Roche, Mannheim, Germany) under the conditions recommended by the manufacturer. To validate our results, a random selection of 10% of the samples was re-genotyped for each SNP, and the results were found to be reproducible with no discrepancies noted. Quality-control procedures included call-rate thresholds ≥ 95%, duplicate concordance checks, and Hardy–Weinberg equilibrium assessment within the analytic sample [29,33].

### 4.4. Derived Indices

The TyG index was calculated [18] from fasting triglycerides and glucose as

TyG = ln([TG in mg/dL × Glucose in mg/dL]/2).

This is a validated low-cost surrogate of insulin resistance and has demonstrated relevance for MASLD risk assessment in both pediatric and adult populations, including longitudinal prediction.

The Systemic Immune Inflammation Index (SII) was computed as

SII = (Platelets × Neutrophils)/Lymphocytes (all values in ×10^9^/L units).

SII integrates platelet–neutrophil–lymphocyte dynamics and reflects inflammatory and metabolic stress. Associations with MASLD have been reported in general and adolescent cohorts, although effect shapes (linear vs. U-shaped) may vary across populations [19,23].

### 4.5. Outcomes

The primary outcome was ultrasound-defined hepatic steatosis (binary). Secondary outcomes were continuous metabolic and inflammatory markers, including TG, HDL-c, LDL-c, ALT, AST, GGT, complete blood count, TyG, and SII.

### 4.6. Statistical Analysis

Continuous variables are summarized as mean ± SD (or median [IQR] for non-normal distributions), and categorical variables as number (%). Variable distributions were assessed by visual inspection and Shapiro–Wilk tests. MASLD was coded as a binary outcome (0 = no steatosis, 1 = steatosis) and harmonized prior to analysis.

In genotype–phenotype analyses, for each locus (*PNPLA3*, *MBOAT7*, *MARC1*), associations with continuous traits were evaluated using non-parametric methods to account for non-normality and small subgroup sizes: (a) Kruskal–Wallis tests for additive genotype group comparisons and (b) Jonckheere–Terpstra (J-T) trend tests for ordered allele-dose effects (0, 1, or 2 risk-alleles). JT statistics were calculated using the asymptotic normal approximation with tie correction. One-sided JT tests were applied only when the direction of effect was prespecified based on prior biological and epidemiologic evidence; in all other settings, two-sided testing was used. Because genotype–phenotype analyses were exploratory, results are interpreted descriptively. Key conclusions were unchanged when two-sided testing was applied, and no findings remained statistically significant after correction for multiple comparisons.

Where biologically justified and cell counts permitted, dominant or recessive genetic models and preplanned two-group allele-burden contrasts (e.g., 0 + 1 vs. 2; 0 vs. 1 + 2) were evaluated. For two-group comparisons, the JT test reduces to a one-sided Mann–Whitney test; Kruskal–Wallis H statistics are reported for completeness.

The cumulative burden score was defined a priori using *PNPLA3* (G) and *MBOAT7* (T) as established risk-increasing alleles. Consistent with prior literature, *MARC1* exhibits effects in the opposite direction to *PNPLA3* and *MBOAT7*. The A-allele (threonine) is protective, while the G-allele (alanine) represents the non-protective state; accordingly, the G-allele was incorporated into the analysis together with the risk alleles of *PNPLA3* and *MBOAT7*. Therefore, the primary burden model coded *MARC1* (G) as the risk-increasing allele. A three-locus cumulative risk-allele score was computed across *PNPLA3* (G), *MBOAT7* (T), and *MARC1* (G). Associations with continuous metabolic and hematologic inflammatory indices (e.g., TyG, SII, lipids, liver enzymes) were assessed using Kruskal–Wallis and JT tests, while MASLD prevalence across burden strata was compared using χ^2^ or Fisher’s exact tests, as appropriate.

To account for potential confounding, multivariable regression models were used to assess independent genetic effects. Linear regression models adjusted for age and BMI were applied for continuous outcomes, and logistic regression models adjusted for age and BMI were used for MASLD status. Because of potential collinearity among metabolic variables (e.g., glucose, lipids, TyG), variance inflation factors were calculated, and only models with acceptable collinearity were reported. After adjustment, no individual variant or cumulative genetic burden showed an independent association with MASLD or related traits.

Complete-case analysis was applied to each outcome; genotype–trait comparisons with fewer than two observations per cell were excluded to avoid unstable estimates. Additional exploratory stratifications by BMI category and TyG tertiles were conducted to evaluate potential effect modification. Sensitivity analyses included (i) alternative *MARC1* coding (protective vs. risk); (ii) cumulative burden models excluding *MARC1*, and (iii) stratification by BMI category or TyG tertiles to evaluate potential effect modification.

Current clinical guidance emphasizes non-invasive evaluation in MASLD, including ultrasound for steatosis, and sequential serologic or imaging-based tools (e.g., FIB-4, elastography/ELF) for fibrosis assessment, particularly in populations with cardiometabolic risk [31,32]. The inclusion of TyG (insulin-resistance surrogate) and SII (as a composite inflammatory index) aligns with the metabolic-inflammatory framework of MASLD and emerging pediatric evidence supporting their diagnostic and prognostic relevance [22,23]. All statistical analyses were conducted using SPSS, Version 29.0.0.0.

## 5. Conclusions

In this female-only cohort of late-adolescent participants, ultrasound-defined MASLD was present in 16.7%, within the expected range for general adolescent populations. Across the examined loci (*PNPLA3*, *MBOAT7*, *MARC1*), genotype-wise comparisons did not yield statistically significant associations with routine biochemical markers. Directional, descriptive patterns were observed, with higher MASLD prevalence among *PNPLA3* G- and *MBOAT7* T-allele carriers and a non-uniform, directionally favorable pattern for *MARC1* A165T, although these trends should be interpreted cautiously given modest subgroup sizes.

Cumulative genetic burden analyses demonstrated nominal, unadjusted trends for triglycerides and the TyG index, which were not retained after adjustment for age and BMI. Similarly, the TyG index and the SII exhibited substantial inter-individual variability but did not demonstrate independent predictive value for MASLD in this relatively healthy, low-obesity adolescent cohort.

Overall, these findings highlight modest, exploratory genotype-related patterns in metabolic and hematological immune-inflammation indices during late adolescence rather than definitive evidence of early genetic susceptibility or clinically actionable risk stratification. Larger, longitudinal, female-focused studies incorporating advanced imaging modalities are required to clarify the temporal relationships between genetic variation, metabolic-inflammatory profiles, and MASLD development in adolescence.

This design—focused on females aged 16–19 years with lower obesity burden—addresses early susceptibility rather than advanced disease in mixed-sex cohorts. To our knowledge, this is the first study combining *PNPLA3*, *MBOAT7*, and *MARC1* with TyG and SII exclusively in late-adolescent females. The findings suggest that sex-specific genetic, metabolic, and hematological immune-inflammation variation may be relevant to early MASLD susceptibility in late-adolescent females, but this requires validation in larger longitudinal studies with advanced imaging and multi-omics to refine precision-based risk models for adolescent MASLD.

## Figures and Tables

**Table 1:** Baseline characteristics of adolescent females (*n* = 150).

Variable	*n*	Mean ± SD	Median	Min–Max
Age (years)	150	16.67 ± 0.86	16.00	16.00–19.00
BMI (kg/m^2^)	150	23.64 ± 3.92	22.91	14.70–38.62
Glucose (mmol/L)	150	4.66 ± 0.43	4.65	3.30–6.90
Total Cholesterol (mmol/L)	150	4.16 ± 0.69	4.12	2.80–7.05
HDL-c (mmol/L)	150	1.57 ± 0.33	1.54	0.92–2.62
LDL-c (mmol/L)	150	2.22 ± 0.63	2.14	0.73–4.67
Triglycerides (mmol/L)	150	0.83 ± 0.34	0.75	0.00–2.31
ALT (U/L)	150	17.75 ± 7.70	15.00	8–53
AST (U/L)	150	20.29 ± 5.57	19.00	11–53
GGT (U/L)	150	11.99 ± 3.49	12.00	4–24
Platelets (×10^9^/L)	150	314.81 ± 57.09	310.00	193–482
WBC (×10^9^/L)	150	7.64 ± 1.83	7.43	4.13–12.53
Neutrophils (×10^9^/L)	150	4.48 ± 1.53	4.26	1.68–8.31
Lymphocytes (×10^9^/L)	150	2.37 ± 0.58	2.33	0.91–4.57
CRP (mg/L)	150	1.51 ± 2.10	0.60	0.30–9.99
TyG	150	10.66 ± 0.69	10.56	9.07–12.84
SII	150	631.74 ± 314.72	560.71	144.36–2459.11
Overweight/Obesity, *n* (%) *	150	48 (32.0%)	/	/
MASLD, *n* (%) ^#^	150	25 (16.7%)	/	/

Values are presented as mean ± SD, median, and range (min–max), as appropriate. Abbreviations: ALT, alanine aminotransferase; AST, aspartate aminotransferase; BMI, body mass index; CRP, C-reactive protein; GGT, gamma-glutamyl transferase; HDL-c, high-density lipoprotein cholesterol; LDL-c, low-density lipoprotein cholesterol; MASLD, metabolic dysfunction-associated steatotic liver disease; SII, systemic immune-inflammation index; TyG, triglyceride–glucose index; WBC, white blood cell count. * Overweight/obesity was defined as BMI ≥ 25 kg/m^2^. ^#^ MASLD was defined as ultrasound-detected hepatic steatosis in the presence of at least one cardiometabolic risk factor.

**Table 2:** Metabolic and inflammatory phenotypes by genotype in a female late-adolescent cohort.

	^$^ Phenotype	*PNPLA3* CC/CG/GG	*MBOAT7* CC/CT/TT	*MARC1* AA/AG/GG	*K-W H*	K-W *p*	JT Z	JT Trend *p*
**Metabolic Markers**	**GGT (U/L)**	11.57/12.33/12.77	11.69/11.84/12.74	12.44/11.63/12.23	4.79	PNPLA3: 0.09	2.16	PNPLA3: 0.02 *
	**TyG**	10.62/10.68/10.77	10.55/10.65/10.83	10.53/10.59/10.76	5.02	MBOAT7: 0.08	2.30	MBOAT7: 0.01 *
	**TG (mmol/L)**	0.80/0.86/0.87	0.80/0.82/0.88	0.77/0.82/0.86	4.19	MBOAT7: 0.12	2.10	MBOAT7: 0.02 *
	**LDL** **-** **c (mmol/L)**	2.24/2.23/2.11	2.05/2.34/2.24	2.36/2.17/2.24	5.82	MBOAT7: 0.05	1.31	MBOAT7: 0.10
	**ALT (U/L)**	17.4/18.4/16.8	17.1/18.2/17.7	18.2/17.4/18.0	/	all ns	/	all ns
	**AST (U/L)**	20.5/20.1/20.2	20.1/20.5/20.1	20.3/20.1/20.5	/	all ns	/	all ns
**Inflammatory Indices**	**SII**	596/672/639	619/583/746	560/635/645	3.99	MBOAT7: 0.14	1.28	MBOAT7: 0.10
	**Eosinophils (%)**	1.95/1.64/2.26	1.43/1.77/2.59	1.74/1.67/2.04	3.92	MBOAT7: 0.14	1.88	MBOAT7: 0.03 *
**Other**	**BMI (kg/m^2^)**	23.35/23.99/23.63	23.81/23.24/24.20	24.6/23.5/23.5	/	all ns	/	all ns
*^#^* ** Gene (SNP)**		**β (SE)**	**OR (95% CI)**	***p*** ** Value**				
***MBOAT7 *** **rs641738**		0.85 (1.11)	2.33 (0.27–20.36)	0.44				
***MARC1 *** **rs2642438**		−0.30 (1.14)	0.74 (0.08–6.93)	0.79				
***PNPLA3 *** **rs738409**		9.00 (7.08)	8112.8 (0.01–8.65 × 10^9^)	0.20				

**^$^** *p*-values correspond to Kruskal-Walis (K-W *p*) and trend Jonckheere–Terpstra tests for ordered allele-dose effects (JT Trend *p*). Statistically significant results (*p* < 0.05) are shown in bold and asterisk (*). “ns” indicates non-significant results. *^#^* Multivariable linear regression adjusted for age and BMI under an additive genetic model. Values are presented as β (SE), odds ratios (OR) with 95% confidence intervals (CI), and *p*-values. Abbreviations: ALT, alanine aminotransferase; AST, aspartate aminotransferase; BMI, body mass index; CI, confidence interval; GGT, gamma-glutamyl transferase; JT, Jonckheere–Terpstra test; K-W, Kruskal–Wallis test; LDL-c, low-density lipoprotein cholesterol; OR, odds ratio; SE, standard error; SII, systemic immune-inflammation index; TG, triglycerides; TyG, triglyceride–glucose index.

**Table 3:** Association between selected genetic variants and MASLD after multivariable logistic regression adjusted for age and BMI.

Section	Predictor	N	β (SE)	OR (95% CI)	*p* Value
SNP-specific age- and BMI-adjusted models	*MBOAT7* rs641738	150	2.168 (1.741)	8.74 (0.29–265.52)	0.213
SNP-specific age- and BMI-adjusted models	*MARC1* rs2642438	150	−2.762 (3.411)	0.06 (0.00–50.62)	0.418
SNP-specific age- and BMI-adjusted models	*PNPLA3* rs738409	150	9.00 (7.080)	8112.8 (0.01–8.65 × 10^9^)	0.200
Parsimonious clinical model	Age (years)	150	1.511 (1.547)	4.53 (0.22–93.97)	0.329
Parsimonious clinical model	BMI (kg/m^2^)	150	9.823 (5.694)	18,457.36 (0.26–1,296,755,621.54)	0.084
Parsimonious clinical model	Glucose (mmol/L)	150	−0.627 (3.393)	0.53 (0.00–413.24)	0.853
Parsimonious clinical model	GGT (U/L)	150	0.186 (0.341)	1.20 (0.62–2.35)	0.585

Binary logistic regression models for ultrasound-defined MASLD. SNP-specific models were adjusted for age and BMI. A parsimonious clinical model included age, BMI, glucose, and GGT after evaluation of collinearity. Statistical Regression Model: MASLD ~ genotype_dosage + age + BMI. Abbreviations: BMI, body mass index; GGT, gamma-glutamyl transferase; MASLD, metabolic dysfunction-associated steatotic liver disease; OR, odds ratio; CI, confidence interval; SE, standard error.

## Data Availability

The original contributions presented in this study are included in the article/Supplementary Materials. Further inquiries can be directed to the corresponding authors.

## Supplementary Materials

The following supporting information can be downloaded at: https://www.mdpi.com/article/10.3390/ijms27114837/s1.

## Abbreviations

The following abbreviations are used in this manuscript:

ALT	Alanine aminotransferase
AST	Aspartate aminotransferase
BMI	Body mass index
CRP	C-Reactive protein
GGT	Gamma-glutamyl transferase
HDL-c	High-density lipoprotein cholesterol
LDL-c	Low-density lipoprotein cholesterol
MARC1	Mitochondrial Amidoxime Reducing Component 1
MASLD	Metabolic dysfunction-associated steatotic liver disease
MBOAT7	Membrane-Bound O-Acyltransferase Domain-Containing Protein 7
PNPLA3	Patatin-Like Phospholipase Domain-Containing Protein 3
SII	Systemic immune-inflammation index
SOD2	Superoxide Dismutase 2
TG	Triglycerides
TyG	Triglyceride-glucose index
WBC	White blood cells
