# Supplementary material for: Association of Genetic Variants in PNPLA3, MBOAT7, and MARC1 with Metabolic and Hematological Immune-Inflammation Indices in Adolescent Females with and Without MASLD: A Multilevel Risk-Allele Burden Analysis
Source: Int J Mol Sci. 2026 May 27;27(11):4837. doi: 10.3390/ijms27114837 (PMC13256969; doi:10.3390/ijms27114837)

Supplementary Materials:

Table S1. Pediatric genetic MASLD studies: design contrasts with the present cohort.

**Study**	**Population & Age**	**Sex**	**Obesity / MASLD %**	**Phenotypes & endpoints**	**Multiple testing**	**Relevance for early adolescent MASLD**
Stasinou et al., Nutrients 2022	91 Greek children/adolescents; 3–16 y; 3 groups by weight/MASLD	Both sexes; balanced	High obesity; MASLD ≈56%	MASLD/NASH (US/biopsy), BMI, BP, HOMA-IR; lipids	No correction (α=0.05)	Mixed sex; broader/younger age; disease-enriched; early-risk lens limited
Mansoor et al., PGHN 2021	126 obese children; 5–18 y; 84 MASLD, 42 controls	Both sexes (~55% boys)	100% obese; MASLD 67%	Genotype vs MASLD (US+ALT/AST), enzymes, lipids; GLM/Logistic	No correction	Obesity-enriched; mixed sex; focus on case–control MASLD, not early risk
Nobili et al., Genes & Nutrition 2014	200 suspected MASLD; median 11 y	Both sexes (56% girls)	Obesity 58%; MASLD ~99%	US steatosis; BMI/WC, lipids, GGT; diet/activity interactions	No correction	Younger age; mixed sex; near-universal MASLD—advanced clinical spectrum
Hudert et al., 2022	≈1000 pediatric MASLD cohort (biopsy subset)	MASLD boys 66.6% vs controls 43.9%	MASLD 59% overall	*PNPLA3, TM6SF2, HSD17B13, MARC1*; regression; multi-omics	FDR used (scope unclear)	Large, mixed-sex, fibrosis-oriented; not focused on early adolescent girls
Al-Serri et al., J Hepatol 2012	71 pediatric fibrotic trios; 502 adult biopsy MASLD	Adults 67.5% male; pediatric sex NR	Advanced MASLD (fibrosis)	*SOD2 rs4880; PNPLA3* as covariate; fibrosis severity	No correction	Low relevance to early onset; pediatric arm = fibrotic MASLD only
Present cohort (Slovenia, 2026)	150 late-adolescent females; 16–19 y; population-based	Females only (100%)	Obesity ≈ 8.7%; MASLD = 16.7%	Ultrasound steatosis; BMI/WWI/OS; lipids; liver enzymes; TyG; SII; oxidative-stress panel; *PNPLA3, MBOAT7, MARC1, SOD2*	FDR (BH) applied in Supplement; nominal p-values shown in main text	Narrow, female-only 16–19 y window; low-obesity, early-stage cohort ideal for evaluating subclinical susceptibility and gene–metabolic–inflammatory interactions

Abbreviations: ALT, alanine aminotransferase; AST, aspartate aminotransferase; BMI, body mass index; BP, blood pressure; FDR, false discovery rate; GGT, gamma-glutamyl transferase; GLMM, generalized linear mixed model; HOMA-IR, homeostatic model assessment of insulin resistance; MASLD, metabolic dysfunction-associated steatotic liver disease; NASH, metabolic dysfunction-associated steatohepatitis; SNP, single nucleotide polymorphism; SII, systemic immune-inflammation index; US, ultrasound; WC, waist circumference.

Table S2. Definitions of Metabolic, and Inflammatory Indices Assessed in This Study.

Metabolic and Obesity-Related Indices

Parameter	Full Name	Meaning	Formula
HIS	Hepatic Steatosis Index	NAFLD predictor	$8 \times (ALT/AST) + BMI$
FLI	Fatty Liver Index	Fatty liver predictor	$(e\wedge(0.953lnTG + 0.139BMI + 0.718lnGGT + 0.053WC - 15.745))/(1 + e\wedge(same)) \times 100$
WWI	Weight-Adjusted Waist Index	Central obesity	$WC/\sqrt{Weight}$
AIP	Atherogenic Index of Plasma	CVD risk	$log_{10}(TG/HDL - C)$
TyG	Triglyceride-Glucose Index	Insulin resistance	$ln[TG \times Glucose/2]$
METS-IR	Metabolic Score for IR	Insulin resistance	$ln(2 \times FPG + TG) \times BMI/ln(HDL - C)$
TyG-BMI	TyG-BMI Index	IR surrogate	$TyG \times BMI$
TyG-WC	TyG-Waist Circumference	Visceral adiposity	$TyG \times WC$
TyG-WHtR	TyG-Waist-to-Height Ratio	Central obesity	$TyG \times (WC/Height)$

Inflammation-Related Indices

Parameter	Full Name	Meaning	Formula
MHR	Monocyte-to-HDL Ratio	Chronic inflammation	$Monocytes/HDL - C$
SII	Systemic Immune-Inflammation Index	Immune-inflammatory status	$Neutrophils \times Platelets/Lymphocytes$

Table S3. Age- and BMI-adjusted linear regression models for continuous metabolic and inflammatory outcomes.

Outcome	N	PNPLA3 rs738409 β (SE)	PNPLA3 rs738409 p	MBOAT7 rs641738 β (SE)	MBOAT7 rs641738 p	MARC1 rs2642438 β (SE)	MARC1 rs2642438 p
Glucose	150	0.042 (0.055)	0.445	0.088 (0.049)	0.074	-0.088 (0.053)	0.099
Total Cholesterol	150	-0.053 (0.088)	0.550	0.097 (0.079)	0.220	-0.096 (0.085)	0.263
HDL-c	150	-0.056 (0.041)	0.177	-0.010 (0.038)	0.792	-0.021 (0.041)	0.604
LDL-c	150	-0.030 (0.080)	0.706	0.101 (0.071)	0.159	0.007 (0.078)	0.931
Triglycerides	150	0.033 (0.041)	0.424	0.032 (0.037)	0.397	-0.058 (0.040)	0.146
ALT	150	-0.064 (0.938)	0.946	0.637 (0.842)	0.451	-0.156 (0.914)	0.864
AST	150	-0.306 (0.706)	0.665	0.037 (0.635)	0.953	-0.239 (0.688)	0.728
GGT	150	0.529 (0.406)	0.195	0.575 (0.364)	0.116	-0.218 (0.397)	0.584
CRP	150	-0.154 (0.239)	0.520	0.152 (0.215)	0.480	0.295 (0.232)	0.207
TyG	150	0.046 (0.085)	0.590	0.131 (0.076)	0.087	-0.156 (0.083)	0.062
SII	150	41.832 (40.187)	0.300	63.882 (35.877)	0.077	-29.336 (39.215)	0.456

Multivariable linear regression assuming an additive genetic model. Values are presented as β (SE) and p values. Models were adjusted for age and body mass index (BMI). Sex was not included as a covariate because the analytic cohort comprised females only. Abbreviations: ALT, alanine aminotransferase; AST, aspartate aminotransferase; BMI, body mass index; CRP, C-reactive protein; GGT, gamma-glutamyl transferase; HDL-C, high-density lipoprotein cholesterol; LDL-C, low-density lipoprotein cholesterol; SE, standard error; SII, systemic immune-inflammation index; TyG, triglyceride–glucose index.

Figure S1. Association between the triglyceride–glucose (TyG) index and a cumulative genetic risk-allele score.

(a) Continuous allele-count model (0–6 risk-alleles) using PNPLA3 (G), MBOAT7 (T), and MARC1 (G, corresponding to the non-protective allele; the T-allele is protective). Box-and-whisker plots show the interquartile range (IQR) with the median line; whiskers extend to 1.5×IQR; circles denote outliers. The x-axis label explicitly lists the risk-increasing allele at each locus used in the count. (b) Three-level allele-burden model (Low 0–1, Intermediate 2–3, High ≥4). Plot elements as in panel (a).

(a)

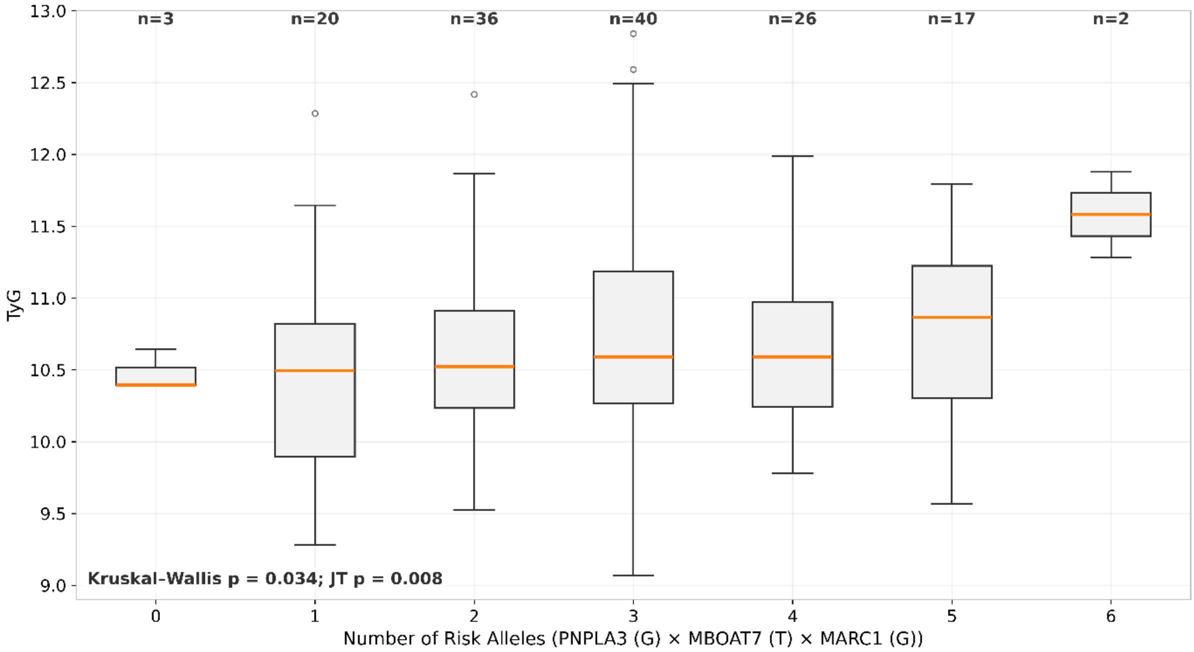

(b)

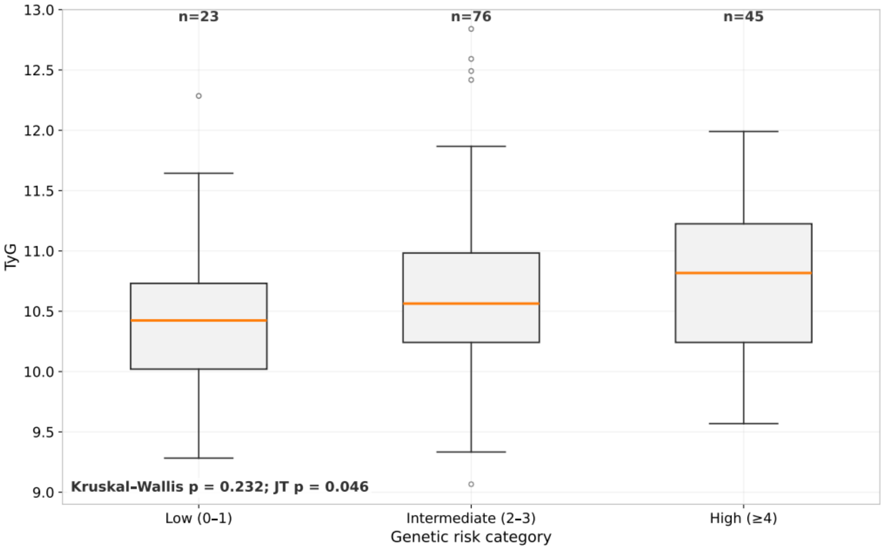

Figure S2. Association between the systemic immune-inflammation index (SII) and a cumulative genetic risk-allele score.

(a) Continuous allele-count model (0–6 risk alleles) derived from PNPLA3 (G), MBOAT7 (T), and MARC1 (G, corresponding to the non-protective allele; the T-allele is protective). Box-and-whisker plots display the interquartile range (IQR) with the median line; whiskers extend to 1.5×IQR; circles denote mild outliers. Extreme outliers were removed using Tukey's 3×IQR rule applied to the overall SII distribution. (b) Three-level allele-burden model (Low 0–1, Intermediate 2–3, High ≥4). Plot elements as in panel (a).

(a)

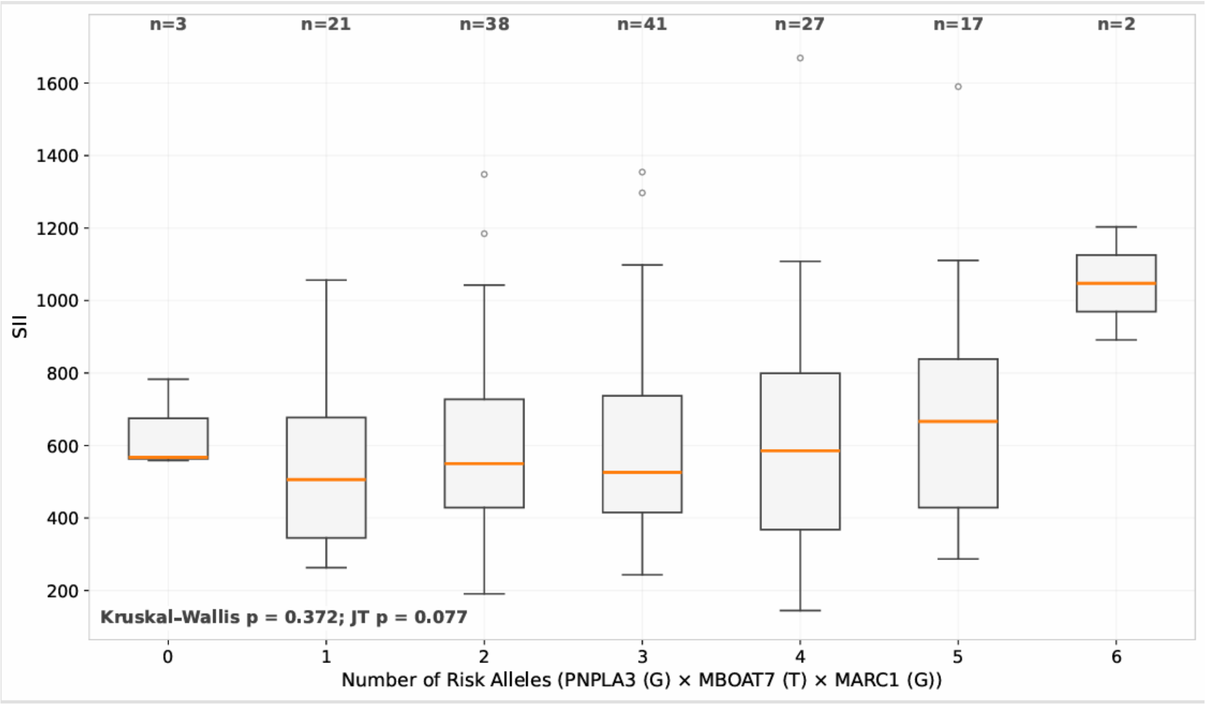

(b)

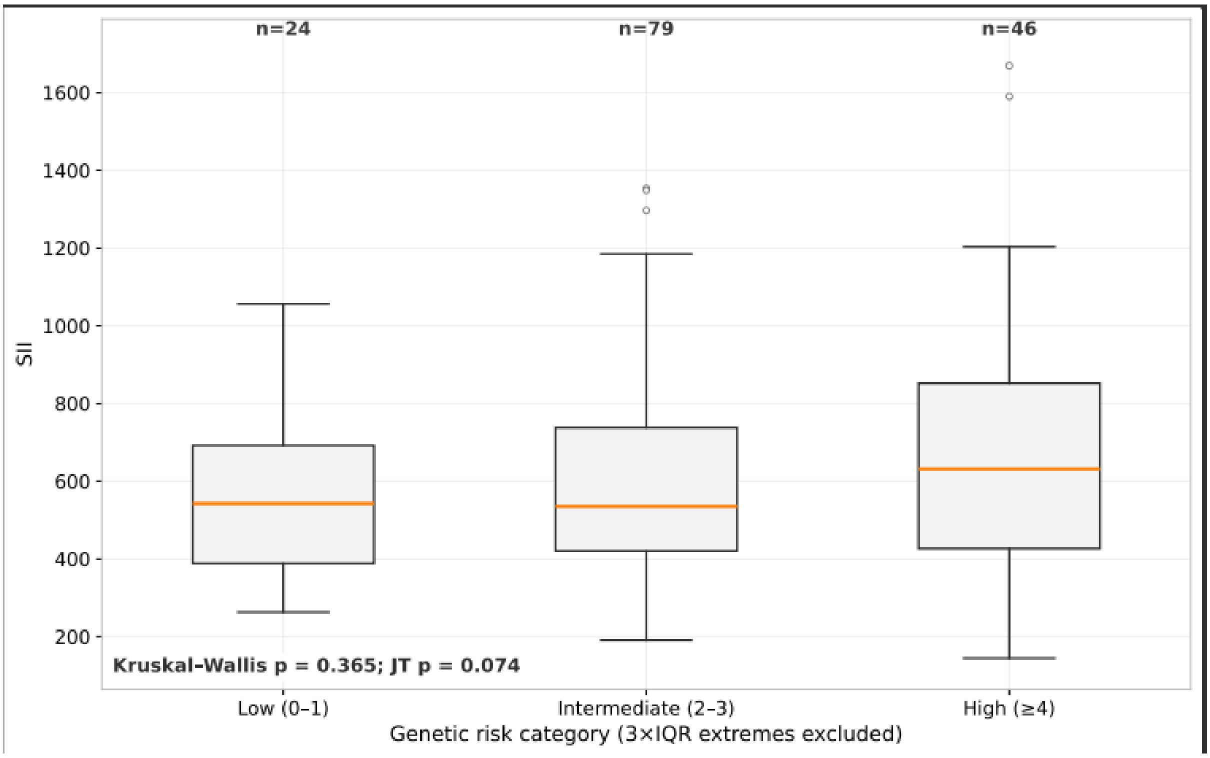

Supplement: Supplementary file 1 [file ijms-27-04837-s001.zip › ijms-4252850-supplementary.pdf]
